# Portable System for Monitoring the Microclimate in the Footwear-Foot Interface

**DOI:** 10.3390/s16071059

**Published:** 2016-07-08

**Authors:** José de Jesús Sandoval-Palomares, Javier Yáñez-Mendiola, Alfonso Gómez-Espinosa, José Martin López-Vela

**Affiliations:** 1 CIATEC A.C, Omega 201, Industrial Delta, 37545 León, Gto., Mexico; jyanez@ciatec.mx (J.Y.-M.); malopez@ciatec.mx (J.M.L.-V.); 2 Tecnologico de Monterrey, Campus Querétaro, Epigmenio González #500, Fracc. San Pablo 76130, Querétaro, Qro., Mexico; agomeze@itesm.mx

**Keywords:** foot, plantar temperature, plantar humidity, thermal monitoring, humidity monitoring

## Abstract

A new, continuously-monitoring portable device that monitors the diabetic foot has shown to help in reduction of diabetic foot complications. Persons affected by diabetic foot have shown to be particularly sensitive in the plantar surface; this sensitivity coupled with certain ambient conditions may cause dry skin. This dry skin leads to the formation of fissures that may eventually result in a foot ulceration and subsequent hospitalization. This new device monitors the micro-climate temperature and humidity areas between the insole and sole of the footwear. The monitoring system consists of an array of ten sensors that take readings of relative humidity within the range of 100% ± 2% and temperature within the range of −40 °C to 123.8 ± 0.3 °C. Continuous data is collected using embedded C software and the recorded data is processed in Matlab. This allows for the display of data; the implementation of the iterative Gauss-Newton algorithm method was used to display an exponential response curve. Therefore, the aim of our system is to obtain feedback data and provide the critical information to various footwear manufacturers. The footwear manufactures will utilize this critical information to design and manufacture diabetic footwear that reduce the risk of ulcers in diabetic feet.

## 1. Introduction

Diabetic foot complications are a particularly global concern because of their clinical outcome and concurrent economic cost [1]. Therefore, international organizations have made it their goal to prevent and reduce such complications through the use of diabetic footwear. The diabetic foot can be defined as the destruction, of varying degrees, of foot tissue that is associated with neurological abnormalities and peripheral vascular disease in the lower limbs [2].

One of most common diabetic disorders located on the plantar surface of the foot is that of dry skin, which may result in fissure formation and eventually in foot ulceration. Foot ulcers are the most common cause of hospitalization and amputation of the lower limb among those with diabetic foot [3]. Other factors [1] that are known to contribute to foot ulcers include lack of sensation, friction, perspiration, and sweating disorder reduction. That the diabetic foot displays certain complications, such as sweating disorders, which results in dry, cracked skin that bleeds [3], could result in fissure formation and, eventually, in foot ulceration [4,5]. Maintaining the skin of the foot in an optimally-hydrated condition helps to prevent the development of fissuring and ulcer formation [6,7,8]. Studies [9,10,11,12] show the combined effects of increasing or decreasing temperature and relative humidity on the behavior of human skin, show an influence of skin hydration and wetness, and some previous studied collected and analyzed the skin changes and the development of superficial ulcer and support surface, considering the microclimate [13,14]. The thermoregulatory sweating abnormality signified early damage in diabetic feet, and assessing skin conditions should help identify neuropathy in diabetic patients with at-risk feet conditions [1].

Since the 1970s there is evidence that the temperature assessment using infrared thermography, contributes to the care of insensitive limbs [15], and that ulcers might by detected by altered temperature of the area; damaged tissue foot areas increases heat on the surface which can be detected and monitored [16]. In this same way, digital thermography experiments using infrared thermal cameras show the association between plantar temperatures and individuals with diabetes [17]. A methodology is presented using infrared thermography and image processing to obtain quantitative temperature differences in the plantar area of the diabetic foot to detect ulceration risks [18].

Studies of microclimate during footwear use are focused in comfort sensations [19] or how low temperature in the ski-boot caused pain sensation, and how comfort is strongly affected by cold feet [20]. An evaluation examined the microclimate in safety footwear and how it may impair specific hygienic parameters, which are critical for wearing comfort and foot health [21], and another study tests the microclimate during footwear use, to obtain the changes in the temperature of the skin of the foot and the temperature and relative humidity of the footwear microclimate for fire fighter footwear [22].

The development of sensor applications as a solution is an important area in healthcare monitoring [23]. It is accepted in literature that it is important to have a quantitative approach to measure temperature and humidity conditions. A monitoring system consisting of a two-dimensional array of fiber optic sensors embedded between two layers of soft, low-loss, and thermally-conductive printed circuit boards (PCB) for a thermal analysis in real-time on the surface of the skin was used in one clinical study [24]. Another development [25] describes an instrumented glove with an array of thermal sensors used to record the human body’s response to thermal conditions in relation to the temperature of the skin and the environment; thus [26,27] present an instrumented in-footwear portable system using independent measuring temperature, humidity, and pressure sensors placed in the insole. TempTouch is a commercial device that reads the surface temperature of the skin using one punctual infrared sensor; allowing the user to monitor his/her temperature. This device was used to conduct a study to identify early signs of vulnerability caused to the diabetic foot. The authors concluded that the temperature monitoring mechanisms were preventive in many cases; as people who underwent traditional foot care control without monitoring temperature were more likely to suffer serious injury or amputation [28].

Nevertheless, there are several studies taking data directly or indirectly about the microclimate in footwear using temperature, relative humidity, and behavior of the skin of the foot, that have not been discussed because they do not completely cover both parameters and how they play a role in diabetic ulcer.

In our work, we developed a continuously-monitoring portable electronic device with one array of 10 sensors to obtain microclimate temperature and humidity data of the foot in the insole of the footwear. By implementing the Gauss-Newton method for the calculation of coefficients [29,30], we developed software to display graphics data of the microclimate. The development was created to perform an analysis of the data’s behavior in a graphical 2D approximation of the data to an exponential curve, as well an exponential response curve. We obtained data from system tests with two male subjects. Preliminary results clearly show that temperature and humidity have an exponential behavior that, as time passes, tends to grow, and then remains constant.

## 2. Materials and Methods

### 2.1. System Description

**Figure 1:**
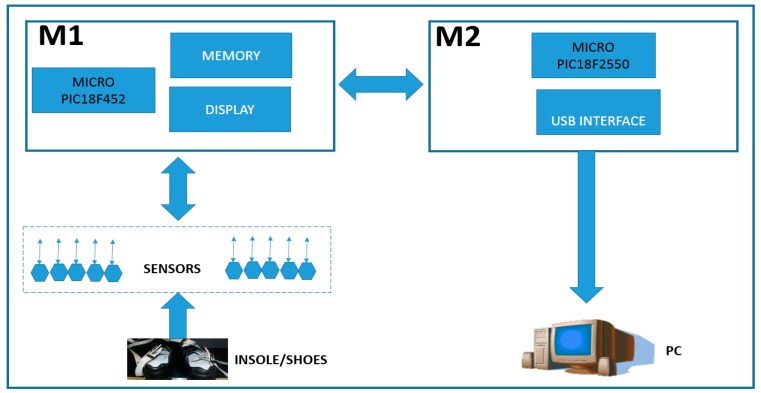
Block diagram of system architecture of portable system for monitoring the footwear-foot microclimate, showing the logical configuration of sensors, microprocessor, memory, USB interface, and PC.

The acquisition of temperature and humidity data is carried out in the microclimate between the plantar surface of the foot and the sole of the footwear, with a system consisting of three modules. The first is a portable module that allows us to gather temperature and humidity data using 10 sensors (M1). After the data is collected, a second module (M2) connects the M1 with a personal computer (PC) utilizing a Universal Serial Bus (USB) interface. The third module is the software interface module (GUI) that allows for acquisition and display of the data in text and graphics. Figure 1 depicts a block diagram of the implemented system.

The M1 architecture consists of ten SHT15 sensors (Sensirion, Stäfa, Switzerland) that are capable of reading ranges of 100% RH ± 2% for relative humidity and −40 °C to 123.8 ± 0.3 °C for temperature in a single chip. For the connection and communication to the printed circuit board (PCB), we used a PIC18F452 microprocessor (Microchip, Chandler, AZ, USA) and AT24C256 memory (Atmel, San Jose, CA, USA). Since the M1 module is autonomous, we are using a commercial 9V battery as our power source. For future microclimate readings, the sensors were divided into two flexible segments of five sensors each and connected to the M1 through an interface wire-board connector header of 14 pins. Additionally, the M1 module includes a display for viewing the temperature and humidity data during capture. Furthermore, the number of readings and total time to read is controlled by a pair of buttons that allows the user to set the time between readings and total number of readings. The link between the M1, M2 and PC is a PIC18F2550 (Microchip, Chandler, AZ, USA) microprocessor that is connected to the M1 through an interface wire-board connector header of 14 pins, while the PC is connected using a USB port.

### 2.2. Software User’s Interface

The temperature and humidity data that has been obtained is stored in a MySQL database. This allows the user to display either stored or current data on the display screen. A Matlab R12 graphical user interface is included to select and plot reading data in 2D. Likewise, it also allows for comparison of data between different sensors, readings intervals, and calculating basic statistics, such as average and standard deviation.

### 2.3. Calculating Curve Behavior

For an analysis of curve behavior, an algorithm was created to determine the least squares for the α and β coefficients of an exponential polynomial (1). In this case, as the curve is not linear, we opted for the use of the Gauss-Newton non-lineal iterative method, where *y* is the dependent variable, *e* is the Euler number, *t* is the time and the α and β coefficients are the calculated independent variables.

(1) $$y=1\;-\;\beta e^{\;\left(-\alpha *t\right)}$$

The data was normalized to present a homogeneous level in the range [0, 1] and for the standardization of the data Equation (2), where *M_i_* is the n-th element of the set *M*, *M_Max_* is the maximum value, and *M_Min_* is the minimum value of the dataset.

(2) $$M_{i}=\;\frac{\left(M_{i}-\;M_{Max}\right)}{\left(M_{Min}-M_{Max}\right)}$$

Equation (1) represents a first-order linear system with an input variable *x* and an output variable *y*, as follows:

(3) $$T\dot{y}+y=\;x$$

In a Laplace field, the transfer function is expressed as follows:

(4) sy(s)+y(s)=x(s)

(5) y(s)(Ts+1)=x(s)

(6) $$\frac{y\left(s\right)}{x\left(s\right)}=\frac{1}{Ts+1}$$

where *T* is the time constant.

Now, obtaining the response to the unit step function (*x*(*s*) = 1/*s*), we have:

(7) $$y\left(s\right)=\frac{1}{Ts+1}\;.\;\frac{1}{s}$$

(8) $$y\left(s\right)=\frac{1}{s}-\;\frac{T}{Ts+1}$$

(9) $$y\left(s\right)=\;\frac{1}{s}-\;\frac{1}{s+1/T}$$

The solution is:

(10) $$y\left(t\right)=1-e^{-t/T}$$

Which, compared with Equation (1), we have:

(11) y(t)=1−βe−αT

where β = 1, α = 1/T, then *T* = 1/α.

For our case, β = 1 and α = 1/*T* define an important independent variable that tells us how fast the answer of the system is.

**Figure 2:**
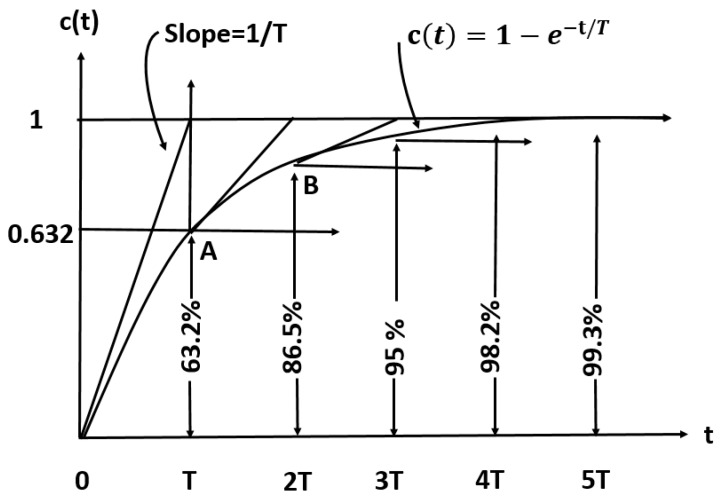
Theoretical exponential response curve.

We try to find the time constant *T* of the exponential response curve of *y*(*t*), from 0% to 63.2%, 86.5%, 95%, 98.2%, and 99.3%; that is, the *T*, 2*T*, 3*T*, and 4*T* time constants of the final value, in correspondence to Figure 2.

### 2.4. Experimental System Test

The sensors were pre-calibrated by the manufacturer, allowing us to proceed testing the data collection validating that the system functioned as expected. To verify autonomy, storage, data transfer, and interface software of the system, tests were performed taking 50, 100, 200, 300, 400, and 500 records.

**Figure 3:**
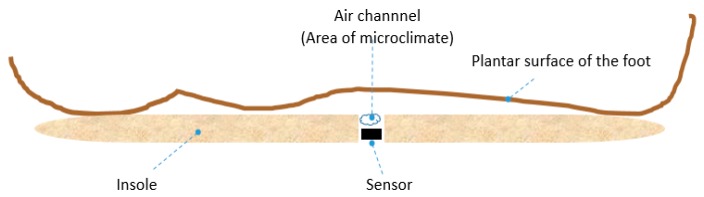
Representative scheme placing sensors in a circular hole in the insole, and the area where the air channel of the microclimate is created, between the plantar surface-insole and the sensor, to read the temperature and humidity.

**Figure 4:**
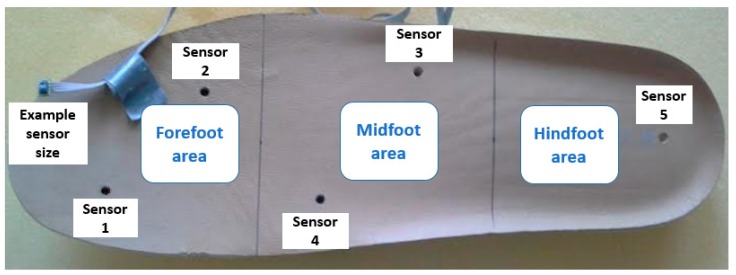
The five sensors were placed in insoles of both the left and right footwear, with the following division of the foot: two sensors for the forefoot, two sensors for the midfoot, and one sensor for the hindfoot.

In our test scenario, we gathered data from two 45-year-old adult male participants who acknowledged being without diseases or pathologies of their feet and in a general healthy condition. An insole 1 cm of thickness was made with five circular holes, followed by placing a sensor in each hole; the holes created an air channel of the microclimate area between the plantar surface-insole and the sensor (Figure 3). This allowed the sensor to read the temperature and humidity data without problems. Figure 4 shows the insole with the placement of the sensors. The five sensors were placed in insoles of both the left and right footwear, with the following division of the foot: two sensors for the forefoot, two sensors for the midfoot, and one sensor for the hindfoot. In a controlled area of 23 °C and 50% relative humidity, insoles were placed inside casual footwear for an acclimatization phase of 5 min. The M1 module was turned on and programmed with reading intervals of 1 min and a data collection duration of 60 min. For the first 5 min, data was gathered from the footwear and insole configurations without feet, followed by the remaining 55 min where participants had their feet in the footwear. After 60 min, the system was turned off and the reading cycle was complete. This testing cycle was repeated five times for each individual. After each cycle, the data was downloaded by connecting the M1-M2-PC and using the GUI software.

## 3. Results

### 3.1. Portable Device

**Figure 5:**
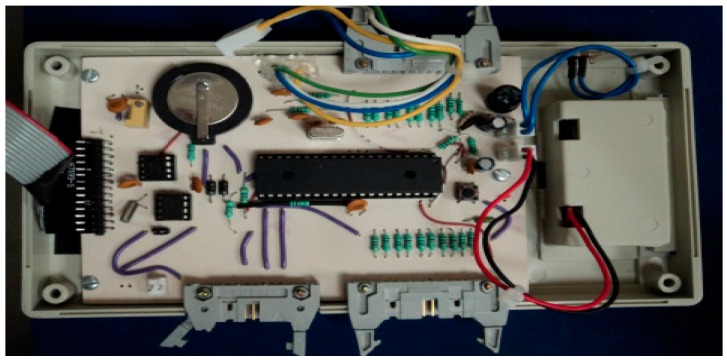
Electronic view of portable system for monitoring the microclimate in the footwear-foot interface.

Figure 5 shows the modules M1 and M2 with their physical components. The size of the M1 case was 5 cm × 7 cm × 3 cm, with a weight of 80 g, including the case, cables, and sensors. The flexible cables are 100 cm long with sensors included; the M1 uses an interface wire-board connector header of 20 pins with a power supply with supply voltages of 2.7 V (2.7 V to 5.5 V) and 1.8 V (1.8 V to 3.6 V). The M1 configuration has two segments with five individual sensors each, with a maximum resolution of 100% RH ± 2% for relative humidity and −40 to 123.8 °C ± 0.3 °C for temperature.

The length of the data record is 128 bytes. With 500 data records of storage needed for 8 h of continued reading at one minute intervals. Furthermore, The M1 has a display of two lines and both a select and enter button to navigate the software menu options.

The display settings are as follows:

- Date and time settings, reading intervals (in minutes), number of readings to be made and data transfer.
- Verification of power display: before starting any new data collection there is a verification of the current battery power. If it is below 2.5 V then the display prompts for a battery.
- The amount of existing data in memory.
- To delete or continue readings option.

**Figure 6:**
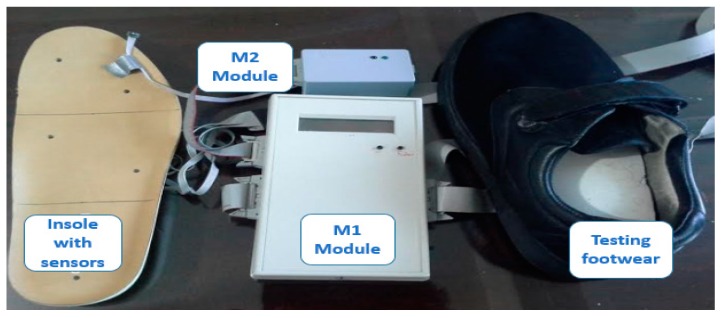
Prototype of the portable system for monitoring the microclimate in the interface of foot, footwear, and insole with sensors.

The screen data displays the detected temperature and humidity and the corresponding readings. Figure 6 depicts the M2 in a portable 2.5 cm × 5 cm × 3 cm case, a wire-board 14 pin connector header interface, and a USB V1.0 connection. The sole purpose of the M2 is to send data from M1 to the PC.

### 3.2. Portable Device

**Figure 7:**
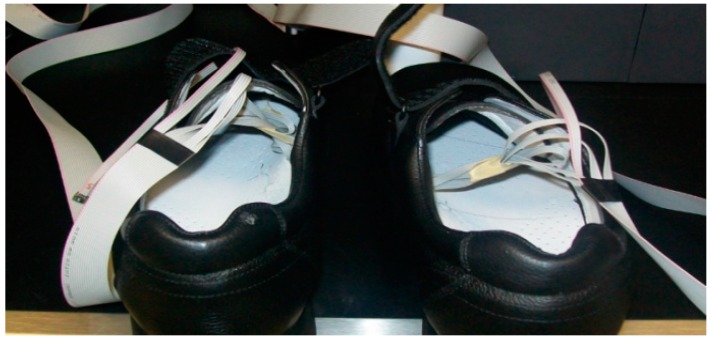
Array of ten sensors placed on flexible cables in the footwear.

**Figure 8:**
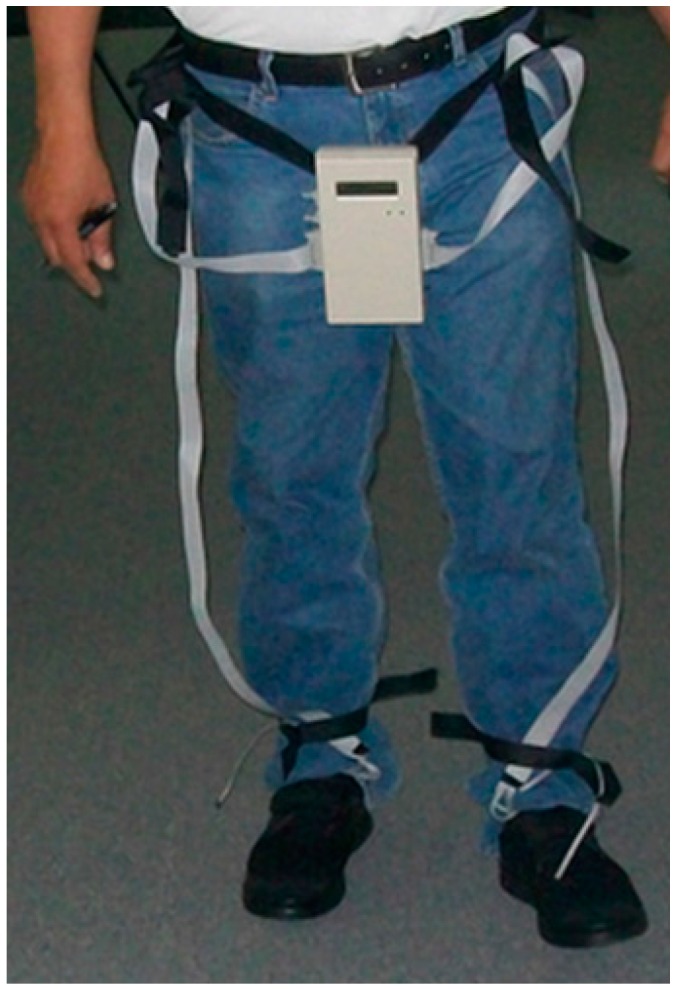
System placed on one of the test subjects.

We performed system validation tests on two healthy men to demonstrate the feasibility of the system. Figure 7 shows the sensors placed in a pair of footwear, while Figure 8 shows the major components of the system placed on one of the test subjects.

There were no technical problems that arose during the 300 temperature and humidity data readings nor during the transfer of the data to the PC. Participants expressed no discomfort in their feet during the experimental validation of the system.

**Figure 9:**
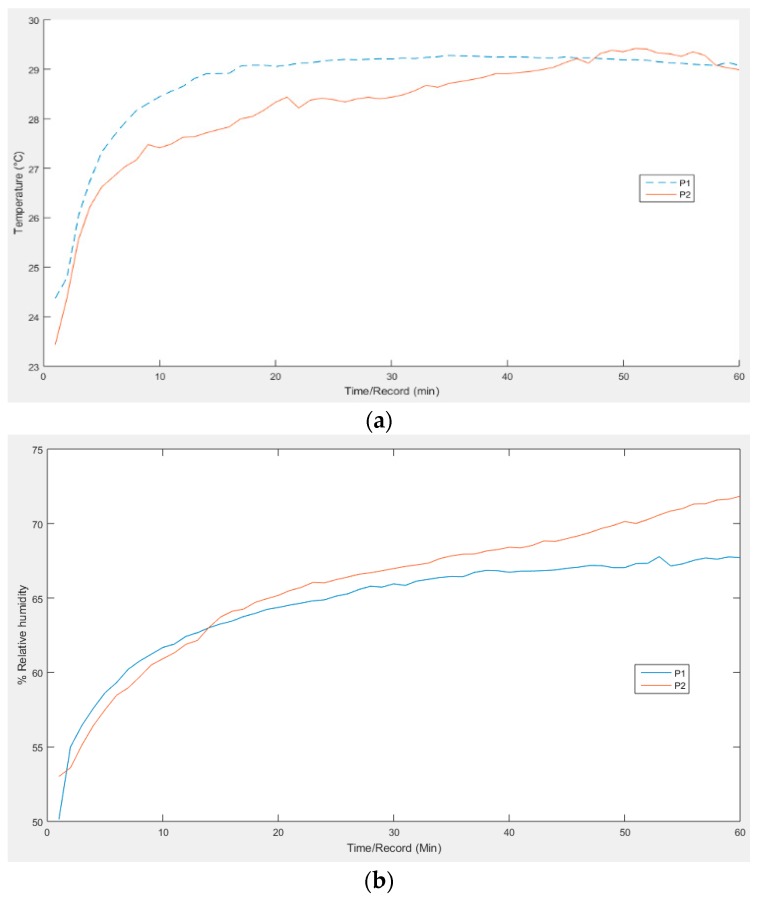
Graph of experimental measurements: (**a**) of temperature; and (**b**) relative humidity. In both temperature and humidity an exponential growth is observed initially.

Temperature monitoring of the participants showed an average range of 24.37 °C to 29.27 °C and 23.44 °C to 29.41 °C. An exponential growth is observed initially, but with a tendency over time for the curve to move horizontally. Around the 40th minute a slight temperature decrease of 0.2 °C is observed, which can be attributed to additional ventilation produced during foot posture change and/or the effect of temperature measurement uncertainties, Figure 9a shows the corresponding experimental readings. In relation to monitoring relative humidity, the results were RH 53.01 to 71.82 and RH 50.13 to 67.75 per participant. Once more, an exponential growth is observed at the beginning, as shown in Figure 9b.

### 3.3. Software Interface

We implemented a Matalab functions menu that is described below:

- Getting function: implements an algorithm to verify that the M2 is connected via USB and requests data collection of temperature and humidity from the M1.
- View function: presents a row-column text data list.
- Analysis function: presents the data in 2D graphics, it is possible to select a particular range reading of interest.

**Figure 10:**
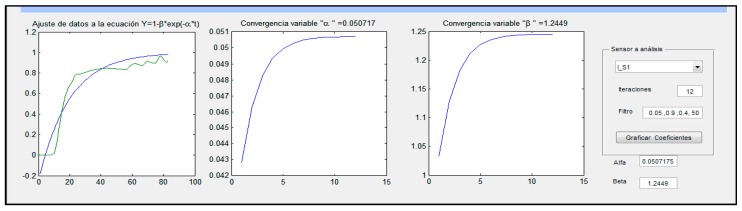
Software interface of to show the example of adjustment of data of temperature and humidity to the exponential curve. This adjustment was made by the nonlinear iterative Gauss-Newton method.

For the following data and defined ranges of interest (63.2%, 86.5%, 95%, 98.2%, and 99.3%), an exponential response curve was found, and adjustment to the resulting exponential curve was made by the nonlinear iterative Gauss-Newton method (see Figure 10).

## 4. Discussion and Conclusions

We provided a microclimate monitoring system with sufficient data storage of temperature and humidity readings for up to eight continuous hours at one minute intervals of portability and autonomy during data collection. The system was designed by placing the sensors in a typical division of the foot (forefoot, midfoot, and hindfoot); its design allows for their placement in other configurations as well. The results demonstrated the technical feasibility of the construction of a portable system, using commercial components at reasonable costs, designed for monitoring temperature and humidity in the footwear-foot interface. In this paper, we conveyed testing results and implemented the iterative Gauss-Newton method to adjust the curve and show the exponential response curve graph.

The results have indicated that the portable system for monitoring microclimates in the foot- footwear interface and in conjunction with the software developed in this work is a useful tool for medical applications that may help decrease or prevent the onset of diabetic ulcers and other diabetic foot complications.

We are planning future work to perform studies using several types of footwear, as well as working with specific population groups that require specialized footwear, such as factory workers and patients with diabetes. This work is limited to 60 readings of temperature and humidity, and in future studies, with different use conditions, longer readings will be considered in order to obtain a more detailed behavior of the microclimate in the footwear-foot interface. We will also consider that the monitored data from this system will help to design footwear in the future, providing information relevant to the best design shape for footwear and the materials from which they should be made, toward the end of manufacturing footwear with agreeable microclimate properties for the control and prevention of ulcers.
